# Non-operating Room Anesthesia (NORA): A Comprehensive Review of Monitored Anesthesia Care

**DOI:** 10.7759/cureus.68024

**Published:** 2024-08-28

**Authors:** Dhruv Shah, Jayshree Sen, Dushyant Bawiskar

**Affiliations:** 1 Anesthesiology, Jawaharlal Nehru Medical College, Datta Meghe Institute of Higher Education and Research, Wardha, IND; 2 Sports Medicine, Abhinav Bindra Targeting Performance, Bangalore, IND

**Keywords:** monitored anesthesia care, midazolam, non-operative, mri, endoscopy

## Abstract

Monitored anesthesia care (MAC) is being increasingly employed in non-operative environments, particularly in the realms of endoscopy and magnetic resonance imaging (MRI) procedures. This in-depth analysis delves into the essential components of MAC within these specific contexts, with a primary focus on ensuring patient safety, evaluating efficacy, and assessing procedural outcomes. It is a common practice in endoscopic procedures to necessitate sedation for the purpose of alleviating discomfort and anxiety, ultimately ensuring patient cooperation and the successful completion of the procedure. MAC, which entails the administration of sedatives and analgesics under the close supervision of an anesthesia professional, offers a personalized approach that carefully balances the depth of sedation with maintaining optimal patient safety standards. Within the domain of MRI procedures, where challenges such as claustrophobia and motion artifacts can significantly impact the process, MAC plays a crucial role in providing a controlled setting that not only enhances image quality but also improves patient compliance throughout the procedure.

The review extensively investigates the various pharmacological agents commonly utilized in these scenarios, including but not limited to midazolam and fentanyl, shedding light on their pharmacokinetic and pharmacodynamic properties specific to these contexts. Furthermore, the critical role of the anesthesia provider in effectively managing potential complications, such as respiratory depression, hemodynamic instability, and allergic reactions, is thoroughly examined and discussed. The analysis extends to the implementation of MAC protocols, encompassing pre-procedural assessments, continuous intra-procedural monitoring, and comprehensive post-procedural care, all aimed at ensuring the best possible outcomes for patients. Additionally, the review delves into the economic considerations associated with MAC, taking into account its impact on procedural efficiency, healthcare costs, and patient throughput within these settings. By exploring current guidelines and recommendations established by professional societies such as the American Society of Anesthesiologists (ASA), this review aims to provide a holistic understanding of the best practices in MAC for both endoscopy and MRI procedures. Through the synthesis of available evidence, the primary objective of this review is to contribute to informing clinical practices, enhancing patient safety measures, improving procedural success rates, and ultimately advocating for the broader adoption of monitored anesthesia care in diverse non-operative medical settings.

## Introduction and background

In recent years, the utilization of monitored anesthesia care (MAC) has witnessed a significant expansion that extends far beyond the confines of traditional operative environments. This growth has been propelled by a multitude of factors, leading to its increasing application in a wide array of non-operative medical procedures. The surge in demand for minimally invasive diagnostic and therapeutic interventions, coupled with the imperative need to enhance patient comfort and safety, has been instrumental in driving this phenomenon. Among the various non-operative settings where MAC has found relevance, endoscopy and magnetic resonance imaging (MRI) stand out as prominent fields that have come to rely on the pivotal role played by MAC [1].

The realm of endoscopic procedures encompasses a diverse range of essential diagnostic and therapeutic tools utilized in modern medical practice. These procedures, which include gastrointestinal endoscopy and bronchoscopy, often necessitate patients to maintain a state of mobility and cooperation throughout the process. However, achieving this state can pose significant challenges due to factors such as discomfort, anxiety, or pain experienced by the patients [2]. While traditional methods such as moderate sedation or conscious sedation have been conventionally employed to address these challenges, their inherent limitations, characterized by variable patient responses and the potential for inadequate sedation, have spurred a growing interest in the application of MAC. Under the supervision of an anesthesia professional, MAC entails the administration of sedatives and analgesics aimed at achieving a deeper and more controlled level of sedation. This approach not only serves to enhance patient comfort but also guarantees safety through continuous monitoring and the ability to address any arising complications promptly [3].

Likewise, the utilization of MAC in MRI settings has gained significant traction, particularly among patients grappling with severe claustrophobia, anxiety, or an inability to maintain stillness for the duration of the imaging study. MRI, being a cornerstone in the realm of diagnosing and managing numerous medical conditions, demands high-quality imaging that is free from motion artifacts. For patients for whom undergoing the procedure without sedation is untenable, MAC emerges as a viable solution that not only fosters patient compliance but also enhances the quality of the resultant images. By administering anesthetic agents under the supervision of an anesthesia provider, precise control over the level of sedation is achieved, thereby reducing movement and optimizing the diagnostic efficacy of the MRI [4].

The pharmacological agents commonly employed in the context of MAC, including midazolam and fentanyl, play an indispensable role in achieving the desired levels of sedation. Propofol, characterized by its short-acting hypnotic properties, is particularly favored for its rapid onset and recovery profile, rendering it ideal for procedures necessitating swift turnaround times. Midazolam, classified as a benzodiazepine, exerts anxiolytic and amnestic effects, whereas fentanyl, an opioid, offers potent analgesic properties. The pharmacokinetics and pharmacodynamics of these agents have been extensively studied, enabling anesthesia providers to tailor sedation protocols in accordance with individual patient requirements and procedural difficulties. A critical facet of MAC pertains to the pivotal role assumed by the anesthesia provider [5]. This professional is tasked with conducting a comprehensive pre-procedural assessment aimed at evaluating the patient's medical history, prevailing medications, and potential risk factors. The insights gleaned from this assessment serve as the foundation for devising a personalized anesthesia plan, encompassing the selection of suitable sedative agents and dosages. Throughout the procedure, the anesthesia provider maintains a continuous vigil over the patient's vital signs, encompassing parameters such as heart rate, blood pressure, oxygen saturation, and respiratory function. This meticulous monitoring regime enables the timely detection and management of any untoward events that may arise, including instances of respiratory depression, hemodynamic instability, or allergic reactions [6].

Economic considerations play a vital role in the discourse surrounding MAC in non-operative environments. The analysis must take into account the initial expenses linked to the employment of anesthesia providers and the utilization of specific anesthetic agents, juxtaposed with the potential cost savings derived from shorter procedure durations, decreased complication rates, and reduced necessity for repeat procedures. Research has indicated that the overall cost-effectiveness of MAC can be advantageous, especially when considering the enhanced patient outcomes and satisfaction that result from its implementation. The most important cost-effective benefit of MAC is that it reduces the utilization of the post-anesthesia care unit (PACU) [7].

The guidelines and recommendations laid out by esteemed professional organizations, such as the American Society of Anesthesiologists (ASA), serve as a blueprint for the safe and efficient integration of MAC. These guidelines stress the significance of meticulous patient selection, comprehensive pre-procedural assessment, vigilant monitoring during procedures, and thorough post-procedural management. Adhering to these optimal practices ensures the safe and effective delivery of MAC, thus minimizing potential risks while maximizing benefits for patients [8,9]. In summary, monitored anesthesia care (MAC) stands out as a substantial progression in the anesthesia field, presenting a secure, efficient, and patient-centric method of sedation in non-operative contexts. Its utilization in endoscopy and MRI procedures serves as a testament to the advantages of this approach, elevating patient comfort levels, procedural effectiveness, and overall results. As the healthcare landscape undergoes continual transformations, the integration of MAC into diverse medical procedures will play a pivotal role in meeting the escalating demand for minimally invasive, top-notch healthcare services [10].

Objective of the review 

Furthermore, this review will delve into the specific duties and obligations of anesthesia providers when implementing MAC protocols, encompassing aspects such as pre-procedural assessments, continuous monitoring during procedures, and post-procedural care to ensure comprehensive patient safety and well-being. The review will also take into account the economic implications of utilizing MAC, including its effects on healthcare expenditure, procedural efficiency, and patient turnover rates. Moreover, it will amalgamate existing guidelines and recommendations put forth by professional medical societies to establish optimal protocols for the safe and efficient application of MAC. In conclusion, the overarching goal of this review is to enrich clinical practice by imparting valuable insights, enhancing patient safety standards, increasing the success rates of procedures, and promoting the wider adoption of MAC in diverse non-operative medical interventions, thereby propelling advancements in the realm of anesthesia care.

## Review

Methodology

Search Strategies

A comprehensive search strategy was deployed in the literature review, involving multiple databases such as PubMed, Scopus, Web of Science, and Google Scholar. This involved "monitored anesthesia care," "non-operative settings," "magnetic resonance imaging," and "endoscopy" as the keywords used. Boolean operators were used to combine search terms and refine search results. Only articles published in English from January 2000 to June 2024 were included in this search to capture recent and relevant studies.

Criteria for Inclusion and Exclusion

Inclusion criteria were set up to identify studies that specifically were based on monitored anesthesia care in non-operative settings, as in the case of endoscopy and magnetic resonance imaging, that had quantitative data on patient outcomes and were peer-reviewed. The exclusion criteria included non-laparoscopic surgical procedures, case reports, review articles, editorials, and studies without full text.

The Extraction of Data and Its Synthesis

A total of 134 articles were initially identified using the search strategy. After removing duplicates, there were 98 articles. These papers were then screened based on titles and abstracts, giving a total of 36 papers for full-text review. Among these many papers that underwent thorough assessment against several inclusion/exclusion criteria, only seven meet methodological standards; hence, they have been considered for detailed analysis. The data extracted from these studies consisted of study design, sample size, patient demographics, ventilation parameters, intraoperative and postoperative outcomes, and major findings contained in the articles themselves. Table 1 represents a description of the articles reviewed for the study.

**Table 1:** Description of studies involved in the review BDE, bidirectional endoscopy; ASA, American Society of Anesthesiologists; EBUS-TBNA, endobronchial ultrasound-guided transbronchial needle aspiration; GEA, gastroepiploic artery; VR, virtual reality

Serial number	Author and year of publication	Participants	Outcome measures	Conclusion
1	Mokahal et al. (2023) [11]	120 consecutive patients were enrolled (60 per arm). Baseline characteristics were similar.	For same-day BDE under monitored anesthesia care (MAC) for differences in the amount of time spent in deep sedation, time to recover from sedation, patient and endoscopist satisfaction, adenoma detection rate, or immediate adverse events between the two groups.	Regarding the duration needed to recover from sedation, the amount of time spent in deep sedation, the satisfaction of patients and endoscopists, the rate of adenoma identification, and adverse events, there are no statistically significant distinctions between the groups. The endoscopy group took less time to reach the cecum, but the other group had higher rates of post-discharge vertigo. The optimal order for same-day BDE can be decided upon individually, taking into account procedure yield, patient risk, and additional variables, including staff exposure and aerosol generation potential.
2	Dhingra et al. (2023) [12]	This single-center, randomized trial included 100 ASA physical status class I-II patients who were scheduled for endoscopic retrograde cholangiopancreatography (ERCP).	Comparing the two groups' mean arterial pressure (MAP) at different times was the main goal; adverse events, induction and recovery times, and oxygen saturation were the supplementary goals.	Etomidate can be suggested for use during ERCP in ASA I/II patients since it provides superior hemodynamic and respiratory stability to propofol.
3	Faruki et al. (2022) [13]	40 adults undergoing hand surgery were randomized to receive either intraoperative VR in addition to MAC or the usual MAC.	The intraoperative propofol dosage per hour (mg · hour-1) was the main result. The length of stay (LOS) in the post-anesthesia care unit (PACU), overall satisfaction, functional outcome, and patient-reported pain and anxiety were among the secondary outcomes.	Without adversely affecting important patient-reported outcomes, VR immersion during hand surgery resulted in considerable decreases in intraoperative propofol dose and PACU LOS.
4	En-Asseri et al. (2024) [14]	The study population consisted of a total of 494 adult patients undergoing elective surgery requiring tracheal intubation.	The study's main finding was the total number of unintended events, such as indications of inadvertently deep or insufficiently mild anesthesia.	The current study found that reducing undesired anesthetic events could not be validated by guiding total intravenous anesthesia alone by routine monitoring, as opposed to using entropy and surgical pleth index. On the other hand, there was a decrease in the amount of propofol used, as well as quicker recovery and post-anesthesia care unit stays.
5	Bhattarai (2009) [15]	The study included 60 patients who were planned to receive monitored anesthesia care during EBUS-TBNA.	Hemodynamic stability: MAC is known to produce fewer hemodynamic disturbances than GEA, which may have an effect on the course of treatment for patients undergoing ERCP operations. Reduced adverse effects from inhalational drugs, procedural efficiency: MAC is linked to faster procedure times, which enhances center efficiency and may have an impact on patient throughput and resource usage. The risk of critical events: GEA is preferred to lower the possibility of essential events relating to compromised ventilation and oxygenation during MAC, emphasizing the significance of patient outcomes and safety.	Compared to remifentanil, dexmedetomidine produced fewer adverse respiratory events during EBUS-TBNA under MAC while maintaining the same overall operating settings. Nonetheless, the use of dexmedetomidine was linked to a postoperative discharge delay.
6	Snyder et al. (2006) [16]	They compared 58 consecutive thyroidectomies performed prior to the study with 58 straight thyroidectomies performed after the study.	The amount of time following surgery that is spent in an outpatient surgical facility before being discharged the same day.	In the patient population under study, thyroidectomies can be carried out under local anesthesia with MAC or general anesthesia, with comparable anticipated outcomes in terms of patient satisfaction, clinical outcomes, and operation results. Additionally, using MAC in conjunction with local anesthesia can shorten the recovery period in an outpatient surgical scenario, thus saving money on medical expenses.
7	Badrinath et al. (2000) [17]	This study involved 100 female outpatients who underwent local anesthesia for breast biopsy operations. The trial was double-masked, randomized, and placebo-controlled.	Reduction in the "rescue" opioid required in a dose-dependent manner.	When used in conjunction with propofol sedation, ketamine significantly reduces the need for further opioids while producing considerable analgesia. During closely managed anesthesia care, the combination of propofol (9.4 mg/mL) and ketamine (0.94-1.88 mg/mL) effectively produces drowsiness and analgesia. At sub-hypnotic dosages, ketamine might be a helpful supplement to propofol sedation.

MAC, also known as monitored anesthesia care, encompasses the provision of sedation and analgesia to individuals undergoing minor surgical interventions or diagnostic examinations. In contrast to general anesthesia, MAC enables patients to maintain a level of consciousness that allows for responsiveness and autonomous breathing. The anesthesia provider is responsible for the continuous monitoring of the patient's vital signs and the adjustment of sedation levels as necessary to ensure both comfort and safety throughout the procedure. The realm of techniques and agents employed in MAC is multifaceted and crucial to the success of the procedure [7]. Sedative agents utilized in MAC include midazolam, a benzodiazepine renowned for its anxiolytic, amnestic, and sedative properties. Dexmedetomidine, an alpha-2 adrenergic agonist, is another essential component offering sedation devoid of respiratory depression. Additionally, opioids such as fentanyl and remifentanil are incorporated for their potent analgesic effects [18].

Monitoring in MAC is a meticulous process that involves the continuous assessment of various parameters to ensure patient well-being. This includes the constant monitoring of heart rate, blood pressure, oxygen saturation, and respiratory rate. Capnography is employed to oversee end-tidal CO_2_ levels, thereby guaranteeing adequate ventilation. Furthermore, electrocardiography (ECG) is utilized for continuous cardiac monitoring to promptly address any deviations from the norm. The techniques employed in MAC are crucial for optimizing patient outcomes and safety. This involves the gradual adjustment of sedative agents to achieve the desired level of sedation tailored to each individual. The use of supplemental oxygen is paramount in maintaining optimal oxygen levels, while proper patient positioning is essential to mitigate risks such as aspiration.

The application of MAC in endoscopy, as well as diagnostic and therapeutic purposes, is a common practice. Various procedures fall under endoscopy, including esophagogastroduodenoscopy (EGD), colonoscopy, and endoscopic retrograde cholangiopancreatography (ERCP). Patient selection criteria for MAC in endoscopy are meticulous, with preference given to individuals falling within the American Society of Anesthesiologists (ASA) physical status I-III category. Those with significant comorbidities or at risk of airway obstruction may necessitate the utilization of general anesthesia for their safety [19].

The benefits of employing MAC in endoscopy are manifold, including reduced recovery times and hospital stays when compared to general anesthesia. Additionally, there is a lower incidence of respiratory complications and hemodynamic instability, contributing to improved patient satisfaction owing to faster recovery and decreased post-procedural grogginess. However, challenges persist in the application of MAC in endoscopy, with a particular focus on maintaining adequate sedation levels without compromising airway patency. The management of potential complications such as hypoxia, hypotension, and aspiration is paramount, as is ensuring seamless communication between the endoscopist and the anesthesia provider to guarantee patient safety and procedural success [20].

The utilization of MAC in the context of MRI procedures proves to be particularly advantageous for individuals who struggle with conditions such as claustrophobia anxiety or have difficulties in maintaining stillness during the imaging process. The selection of patients suitable for MAC in MRI encompasses those individuals who are unable to undergo the MRI procedure without sedation, including those with severe anxiety, claustrophobia, or movement disorders. It is important to note that patients with contraindications to MRI, such as certain implants, may not be deemed appropriate candidates for this modality. The advantages associated with the application of MAC in MRI are manifold. These include the enhancement of patient compliance, which leads to superior-quality imaging outputs and more precise diagnostic outcomes. Furthermore, there is a decrease in the necessity for repeat imaging procedures due to issues related to patient movement or anxiety, alongside an elevation in patient comfort levels and a reduction in stress experienced during the MRI process [9].

However, the integration of MAC in the MRI setting also presents its own set of challenges. These challenges include the requirement for non-ferromagnetic equipment within the MRI environment, thus limiting the selection of monitoring devices that can be employed. Additionally, ensuring patient safety within the confined space of the MRI scanner poses a notable challenge, as does the potential for adverse reactions to contrast agents utilized in certain MRI procedures. From a clinical perspective, several considerations need to be taken into account. These encompass conducting a comprehensive pre-MRI assessment to ascertain the necessity for sedation and identify any possible contraindications. At the same time, effective communication with the MRI technologist is essential to coordinate timing and manage any interruptions that may occur during the imaging procedure [21,22].

In the realm of safety and risk management in the context of MAC procedures, the pre-procedural assessment assumes a critical role. This involves a thorough evaluation of the patient's medical history, encompassing allergies, medication usage, and prior experiences with anesthesia. Furthermore, the identification of risk factors such as obstructive sleep apnea, obesity, and cardiovascular disease is imperative. During the procedure, the continuous monitoring of vital signs is essential, coupled with a readiness to intervene promptly in case of any complications. It is crucial to have emergency equipment and medications readily available to address issues such as airway obstruction, hypotension, and other acute events that may arise. Post-procedural care necessitates monitoring the patient in the recovery area until they meet the specified discharge criteria. Moreover, providing clear post-procedural instructions regarding activity restrictions, medication usage, and follow-up care is crucial to ensure optimal patient outcomes. Emergency preparedness in the context of MAC procedures involves training staff in advanced cardiac life support (ACLS) and emergency airway management, alongside the development of protocols designed for swift responses to adverse events [23].

Furthermore, the safety profile associated with MAC merits attention, as it serves to mitigate the inherent risks linked to general anesthesia, such as respiratory depression and hemodynamic instability. By upholding spontaneous respiration and safeguarding vital airway reflexes, MAC emerges as a safer alternative, especially for patients grappling with concurrent health conditions [24]. Transitioning to the domain of challenges and risk management, several critical aspects warrant consideration. Chief among these is the imperative of maintaining airway patency during MAC administration, particularly evident during endoscopic procedures, where ensuring unobstructed airways poses a significant challenge. Sedative agents utilized in MAC can potentially depress respiratory function, underscoring the need for vigilant monitoring and swift intervention in the event of complications. Similarly, the unique monitoring challenges presented within MRI settings necessitate specialized attention, given the constraints imposed by robust magnetic fields that hinder the use of conventional monitoring equipment. The deployment of MRI-compatible monitors and anesthesia apparatus is imperative, albeit potentially constrained in functionality compared to their standard counterparts [25,26].

Tailoring sedation levels to achieve the optimal balance emerges as a pivotal concern, as both under-sedation and oversedation pose risks. Inadequate sedation may result in patient movement and procedural disruptions, while excessive sedation heightens the likelihood of respiratory and cardiovascular complications. The continuous assessment and judicious titration of sedative agents are, therefore, indispensable to maintaining the delicate equilibrium required for safe and effective sedation. Effective communication and coordination between anesthesia providers and proceduralists are underscored as prerequisites for optimal patient care. This synergy is particularly critical in endoscopy scenarios, where seamless coordination between the two parties is essential for anticipating and responding to patient needs promptly and efficiently [27,28].

Looking toward future directions and innovations in the realm of MAC, advancements in sedative agents play a pivotal role. The development of novel sedative agents with enhanced safety profiles and fewer side effects is a promising area of exploration. Furthermore, the utilization of personalized medicine approaches to tailor sedation protocols according to individual patient genetics and pharmacodynamics holds significant potential for improving patient outcomes. Moreover, the integration of enhanced monitoring technologies represents a notable avenue for innovation [29,30]. This includes the incorporation of noninvasive monitoring devices that offer real-time data on patient physiology, as well as the utilization of artificial intelligence and machine learning algorithms to predict and prevent adverse events during MAC procedures. The exploration of telemedicine strategies for the remote monitoring of patients undergoing MAC in outpatient settings is an emerging area of interest. Additionally, the development of wearable devices that facilitate continuous monitoring and seamless data transmission to healthcare providers is seen as a promising development in the field. Emphasizing patient-centered care is crucial in the context of MAC procedures. This involves a focus on enhancing patient education and communication to improve the overall patient experience. Furthermore, the incorporation of patient feedback to refine sedation protocols and procedural practices is essential for ensuring patient satisfaction and optimal outcomes [19].

Discussion

The application of MAC in non-operative environments, such as endoscopy and MRI, signifies a noteworthy progression in patient healthcare, striking a balance between ensuring patient comfort and procedural efficiency while addressing safety considerations. Within this discourse, a comprehensive analysis is conducted on the pivotal elements of MAC utilization in these particular contexts, exploring its advantages, obstacles, and prospective trajectories. A thorough examination of the advantages of employing MAC in non-operative settings reveals several key benefits that significantly impact patient well-being and procedural outcomes. Firstly, in the realm of enhanced patient comfort and compliance, procedures such as esophagogastroduodenoscopy (EGD) and colonoscopy often evoke substantial discomfort and anxiety among patients [13].

The studies reviewed in this article highlight MAC's benefits, including enhanced patient safety, reduced recovery times, and improved overall satisfaction. For instance, in endoscopy, Mokahal et al. (2023) demonstrated that MAC facilitated safe and efficient procedures without significant differences in patient or endoscopist satisfaction, adenoma detection rates, or adverse events when compared between groups. However, post-discharge vertigo was more common in one group [11]. Similarly, Dhingra et al. (2023) found that using etomidate for MAC during endoscopic retrograde cholangiopancreatography (ERCP) offered superior hemodynamic stability compared to propofol, making it a preferable option for ASA I/II patients [12].

In the context of MRI, the use of MAC has also shown significant promise, particularly for patients with anxiety, claustrophobia, or movement disorders. The reviewed studies support MAC's role in providing effective sedation while maintaining respiratory function and patient safety. Bhattarai (2009) highlighted the advantages of dexmedetomidine over remifentanil in reducing respiratory events during endobronchial ultrasound-guided transbronchial needle aspiration (EBUS-TBNA) under MAC. However, it was associated with a delay in postoperative discharge [15]. Furthermore, Faruki et al. (2022) demonstrated that intraoperative virtual reality (VR) in addition to MAC significantly reduced intraoperative propofol dosage and post-anesthesia care unit (PACU) length of stay without negatively impacting patient outcomes [13]. These findings underscore MAC's potential to enhance procedural efficiency and patient outcomes in non-operative settings while also highlighting the need for the careful management of sedation levels and vigilant monitoring to mitigate risks.

## Conclusions

MAC in non-operative settings, such as those found in endoscopy and MRI procedures, presents a highly compelling combination of factors that contribute to patient well-being, encompassing aspects such as comfort, safety, and the effectiveness of the methods themselves. Despite the existence of certain obstacles that need to be addressed, particularly with regard to the crucial aspects of airway management and monitoring within the MRI setting, it is important to note that continuous progress in technological innovations and the implementation of personalized medicine hold the potential to elevate the quality of care further provided in this context. The successful application of MAC demands a meticulous approach that entails the careful consideration of factors such as patient selection, the customization of sedation protocols to suit individual needs, and the establishment of strong lines of communication among the various members of the healthcare team involved. With the ongoing evolution of this field, it is foreseeable that MAC will progressively establish itself as an essential element within the realm of non-operative procedural care, thereby contributing significantly to the enhancement of patient outcomes and the overall success of such procedures.
